# Evaluation of piezocision and laser-assisted flapless corticotomy in the acceleration of canine retraction: a randomized controlled trial

**DOI:** 10.1186/s13005-018-0161-9

**Published:** 2018-02-17

**Authors:** Alaa M. H. Alfawal, Mohammad Y. Hajeer, Mowaffak A. Ajaj, Omar Hamadah, Bassel Brad

**Affiliations:** 1 Department of Orthodontics, University of Damascus Dental School, Damascus, Syria; 2 Department of Oral Medicine, University of Damascus Dental School, Damascus, Syria; 3 Department of Oral and Maxillofacial Surgery, University of Damascus Dental School, Damascus, Syria

**Keywords:** Acceleration, Piezocision, Laser-assisted, Flapless corticotomy, Minimally invasive surgical procedures, Canine retraction

## Abstract

**Background:**

To evaluate the effectiveness of two minimally invasive surgical procedures in the acceleration of canine retraction: piezocision and laser-assisted flapless corticotomy (LAFC).

**Methods:**

Trial design: A single-centre randomized controlled trial with a compound design (two-arm parallel-group design and a split-mouth design for each arm).

Participants: 36 Class II division I patients (12 males, 24 females; age range: 15 to 27 years) requiring first upper premolars extraction followed by canine retraction.

Interventions: piezocision group (PG; *n* = 18) and laser-assisted flapless corticotomy group (LG; *n* = 18). A split-mouth design was applied for each group where the flapless surgical intervention was randomly allocated to one side and the other side served as a control side.

Outcomes: the rate of canine retraction (primary outcome), anchorage loss and canine rotation, which were assessed at 1, 2, 3 and 4 months following the onset of canine retraction. Also the duration of canine retraction was recorded.

Random sequence: Computer-generated random numbers.

Allocation concealment: sequentially numbered, opaque, sealed envelopes.

Blinding: Single blinded (outcomes’ assessor).

**Results:**

Seventeen patients in each group were enrolled in the statistical analysis. The rate of canine retraction was significantly greater in the experimental side than in the control side in both groups by two-fold in the first month and 1.5-fold in the second month (*p* < 0.001). Also the overall canine retraction duration was significantly reduced in the experimental side as compared with control side in both groups about 25% (*p* ≤ 0.001). There were no significant differences between the experimental and the control sides regarding loss of anchorage and upper canine rotation in both groups (*p* > 0.05). There were no significant differences between the two flapless techniques regarding the studied variables during all evaluation times (*p* > 0.05).

**Conclusions:**

Piezocision and laser-assisted flapless corticotomy appeared to be effective treatment methods for accelerating canine retraction without any significant untoward effect on anchorage or canine rotation during rapid retraction.

**Trials registration:**

ClinicalTrials.gov (Identifier: NCT02606331).

**Electronic supplementary material:**

The online version of this article (10.1186/s13005-018-0161-9) contains supplementary material, which is available to authorized users.

## Background

Prolonged orthodontic treatment duration is considered one of the most challenges that face both patients and orthodontists. Increased treatment time has several side effects such pain, discomfort, caries, white spots formation, gingival recession and apical root resorption [1, 2]. So Several surgical procedures have been introduced to accelerate tooth movement and reduce treatment time. However, corticotomy is considered the most clinically applied and it has been used in various forms over the past two decades [3]. Corticotomy has not been widely accepted by the orthodontic community in spite of its effective in reducing orthodontic treatment time, due to its aggressive nature so that it requires full mucoperiosteal flaps, extensive removal of alveolar cortical bone with the possible post-surgical pain, swelling and hematomas [4, 5].

Therefore, minimally invasive procedures have been recently developed to avoid the disadvantages of invasive corticotomy. These procedures do not require flap raising and they use innovative instruments to decrease the surgical trauma. Some of these minimally invasive surgical procedures are corticision [6, 7], piezocision [8, 9], computer-guided piezocision [10, 11], laser-assisted flapless corticotomy [12, 13], and micro-osteoperforations [14]. The acceleratory effect of minimally invasive corticotomy has been referred initially to regional accelerated phenomena (RAP) which causes transient demineralization and increase cellular activity that is responsible for accelerated tooth movement [15, 16]. Furthermore, selective alveolar decortication can induce the expression of inflammatory markers; raise cytokines levels and osteoclast activity which in turns leads to rapid tooth movement [17, 18].

Corticotomy combined with piezoelectric surgery was introduced in 2007 by Vercelotti and Podesta. Although they recorded a significant reduction of treatment time, this procedure was quite invasive since it required flap elevation and excessive bone removal [19] . In 2009, Dibart et al. developed Piezocision as a minimally invasive technique [8], their procedure was based on small cuts in the buccal gingival to allow the piezosurgery knife to enter and perform cuts in the buccal cortical plate to stimulate the RAP phenomenon, and it also could combine piezocison with selective tunneling when soft or hard tissue grafting is required [20]. Recently, laser was used to perform flapless corticotomy due to its advantages. Seifi in 2012 found that flapless corticotomy accomplished by ER:CR:YSGG Laser accelerated tooth movement in rats [12]. In addition, Salman and Ali reported that perforations performed by Er:YAG Laser have achieved rapid canine retraction in 15 patients [13].

Although it has been claimed that minimally invasive corticotomy can accelerate canine retraction and reduce treatment time, but the scientific evidence about its efficacy is still limited according to a recent published systematic review [21]. And it seems that there is no trial comparing two procedures of minimally invasive corticotomy. Therefore, the primary aim of this study was to evaluate the effectiveness of piezocision versus laser-assisted flapless cortiocotmy in accelerating canine retraction. The secondary aim was to evaluate dentoalveolar changes following canine retraction in terms of ‘molar anchorage loss’ and ‘canine rotation’.

## Methods

### Study design and registration

This study was a randomized single-center controlled trial with a compound design; i.e. a two-arm parallel-group design with a split-mouth design for each arm. There were no changes after trial commencement. This study was approved by the Local Ethics Committee of the University of Damascus Dental School, Syria)UDDS-372-07042015/SRC-2743). This study was registered with ClinicalTrials.gov (https://clinicaltrials.gov;2015: NCT02606331).

### Sample size calculation

Sample size was calculated using Minitab® Version 17 (Minitab Inc., State College, Pennsylvania, USA) assuming that a 0.75-mm (40%) increase in the amount of canine retraction in the surgical side compared to the control side over 1month would be considered clinically significant and taking into account that the variance of this variable from a previous paper was 0.67 [22]. [1] When paired t tests with a significance level of 5% and a power of 85% were imployed, 10 patients at least were required in each group. [2] Using the same assumptions as before but for the sake of comparing the two techniques employing two-sample t tests, 16 patients at least were required in each group. To compensate for any possible attrition (but no more thatn 10%), 18 patients were required for each group.

### Participants and eligibility criteria

Thirty-six adult patients (24 females, 12 males) were enrolled in this trial, they were equally and randomly divided into two groups: piezocision group (PG; *n* = 18) and laser-assisted flapless corticotomy group (LG; *n* = 18). A split-mouth design was employed for each group where the flapless surgical intervention was randomly allocated to one side and the other side served as a control side. Information sheets were distributed to all patients and informed consents were obtained. All patients fulfilled these inclusion criteria: (1) Class II division I patients requiring first upper premolars extraction and two-step retraction technique (2) mild to moderate skeletal class II malocclusion (ANB ≤ 7) (3) overjet greater than 10 mm (4) normal or excessive anterior facial height which was evaluated clinically and radiography through these measures (Y axis, MM, SN-MP) (5) mild crowding ≤3 mm (6) age range between 15 and 27 years with skeletal maturity stage ranging from CS4 to CS6 using the cervical vertebral maturation method proposed by Baccetti et al., 2002 [23] (7) completion permanent dentition (except of third molars) (8) no previous orthodontic treatment (9) healthy patients without systematic diseases that could affect bone and tooth movement and no contraindication (medical or psychological) avoid oral surgery (10) good oral hygiene and healthy periodontium which was evaluated clinically (probing depth ≤ 3 mm, no radiograph evidence of bone loss, plaque and gingival index ≤1 according to Silness and Loe [24](. and the basic characteristics of the sample is given in Table 1.

**Table 1:** Basic characteristics of the sample

	PG	LAFCG	Total sample
Sample Size	18	18	36
Gender (females / males)	11 / 7	13 /5	24 /12
Mean age ± SD (years)	18.70 ± 3.6	17.47 ± 3.3	18.08 ± 3.5
Crowding (no/minimal)	3/15	5/13	8/28
Facial divergence (normal/hyperdivergent)	9/9	8/10	17/19
Posterior crossbite (No /yes)	18/0	18/0	36/0
Overjet increase (moderate/ severe)	6/12	7/11	13/23

*PG* Piezocision group, *LAFCG* Laser-assisted flapless corticotomy group, *SD* standard deviation

### Randomization, allocation concealment and blinding

Simple randomization was conducted by one of the academic stuff (not involved in this research) at the Department of Orthodontics using computer-generated random numbers with an allocation ratio of 1:1. Allocation sequence was concealed using sequentially numbered, opaque, sealed envelopes, which were opened only after the completion of leveling and alignment stage of the dental arches. Blinding of personnel and participants were not applicable. Therefore, blinding was applied only for outcomes’ assessor.

### Leveling and alignment

The orthodontic treatment was conducted by the principal researcher (A.M.H.A.) under the supervision of one of the co-authors (M.Y.H.) at Orthodontic Department of the University of Damscus Dental School. In the beginning, first upper premolars were extracted for all patients, then leveling and alignment was performed using a pre-adjusted orthodontic appliance; MBT 0.022-in. slots size (JISCOP, Gunpo-si, Korea) with the following arch wires sequences: 0,014 in. NiTi or 0.016 in. NiTi (according to the amount of crowding), 0,016 × 0,022 in. NiTi, 0,017 × 0,025 in. NiTi, 0,019 × 0,025 in. Steel which was considered the basal arch wire. Soldered transpalatal arches were placed since the beginning of the treatment as moderate anchorage. After the complement of leveling and alignment, panoramic radiographs were taken to evaluate paralleling of the related teeth roots adjacent to the surgical site (canines and second premolars).

### Surgical intervention

All patients were asked to rinse with Chlorhexidine Gluconate 0.12% for 1 min immediately before the surgical intervention, then local Infiltration was injected in the mucobuccal fold distal to upper canines (Lidocaine HCL 2% - Epinerphrine 1:80,000).Once anesthesia was established, surgical intervention was performed in one side which had been chosen randomly. No subsequent sutures were performed and the surgical side was covered by a piece of Iodoform gauze. All patients underwent the following postsurgical regimen:(1) antibiotic tables (Augmentin: 625 mg Amoxicillin 500 mg + Clavulanate Potassium 125 mg); one tablet three times daily for 1 week, (2) rinses with Chlorhexidine Gluconate 0.12% twice a day for 1 week, (3) ice packs for the first 12 h after the surgery (4) soft diet for 2 days after the surgery (5) analgesics: acetaminophen 500 mg only if necessary (6) nonsteroidal anti-inflammatory drugs were forbidden to avoid overlapping with RAP phenomenon.

#### Piezocision group (PG)

**Fig. 1:**
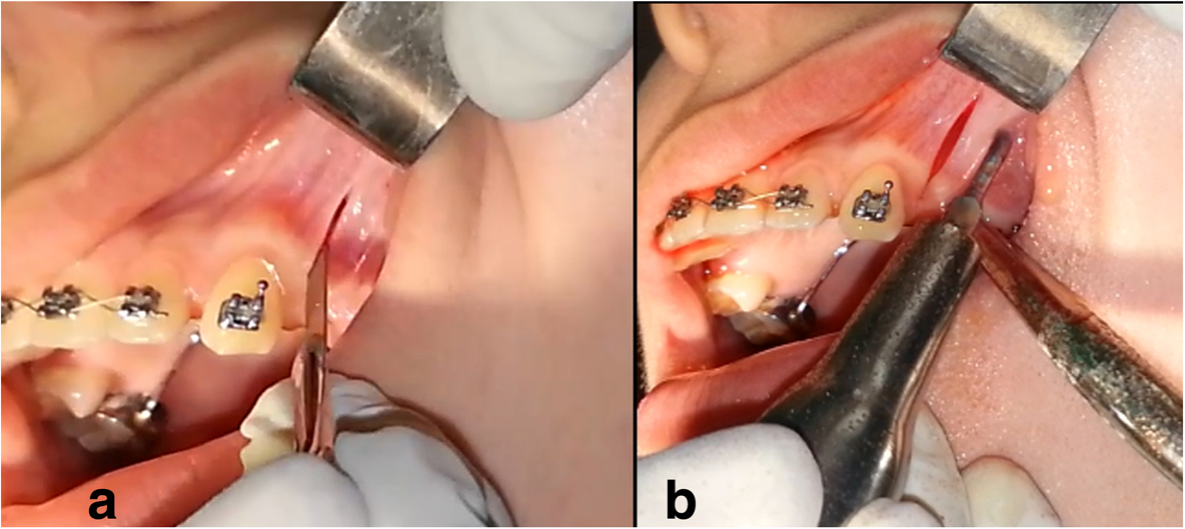
**a**: Soft-tissue incision using blade no 15. **b**: Vertical cortical cuts using a piezosurgery knife

Piezocision was performed by the principal researcher (A.M.H.A) under the supervision of one of the co-authors (B.B.) at Oral and Maxillofacial Surgery Department, University of Damascus Dental School. Two incisions in the buccal gingiva at equal distance from the upper canine and 2nd premolar were done using a surgical scalpel blade N. 15. These incisions started 3-4 mm apical to the interdental papilla and were 10 mm length, then a piezosurgery knife (BS1, Piezotome, Implant Center™ 2, Satelec, France) was inserted to perform alveolar cortical incisions with 3 mm depth, which was confirmed by the millimetric signs on the piezosurgery knife (Fig. 1).

#### Laser-assisted flapless corticotomy group (LG)

**Fig. 2:**
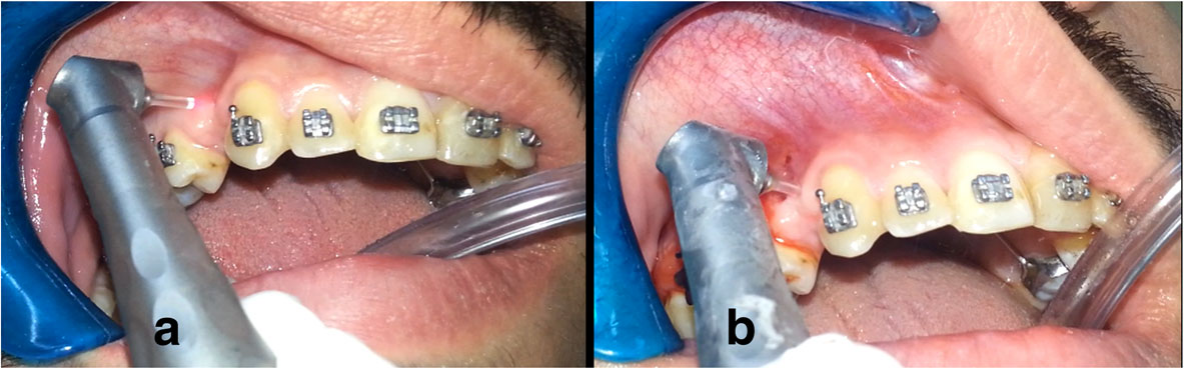
Application of perforations using the ER:YAG laser fiber tip. **a**: Soft-tissue perforations as a first step. **b**: Hard-tissue alveolar cortical perforations

Laser-assisted flapless corticotomy (LAFC) was conducted by the principal researcher (A.M.H.A) under the supervision of one of the co-authors (O.H.) at the Higher Institution for Laser Research and Applications (HILRA), Damascus, Syria. ER: YAG laser (LightWalker® ST-E, Fotona, Ljubljana, Slovenia) was used from with R14C handpiece and Cylinderical Sapphire tip (Diameter: 1,3 mm, Length:8 mm). Five small perforations in the buccal gingiva at equal distance from the upper canine and 2nd premolar were performed using the fiber tip and the device was set at 100 mJ, 10 Hz, 2 W, where each perforation was 1.3-mm wide and away from the other perforation at a distance of 1.5-2 mm. Then the settings were changed to 200 mJ, 12 Hz, 3 W to perform alveolar cortical perforations with 3-mm depth (Fig. 2), which was confirmed with a UNC-15 probe. Both gingival and cortical perforations were performed under water-air spray cooling 40-50 mm/s and with non-contact mode that the fiber tip was 1-2 mm away from the gingiva and the alveolar bone.

### Canines’ retraction

**Fig. 3:**
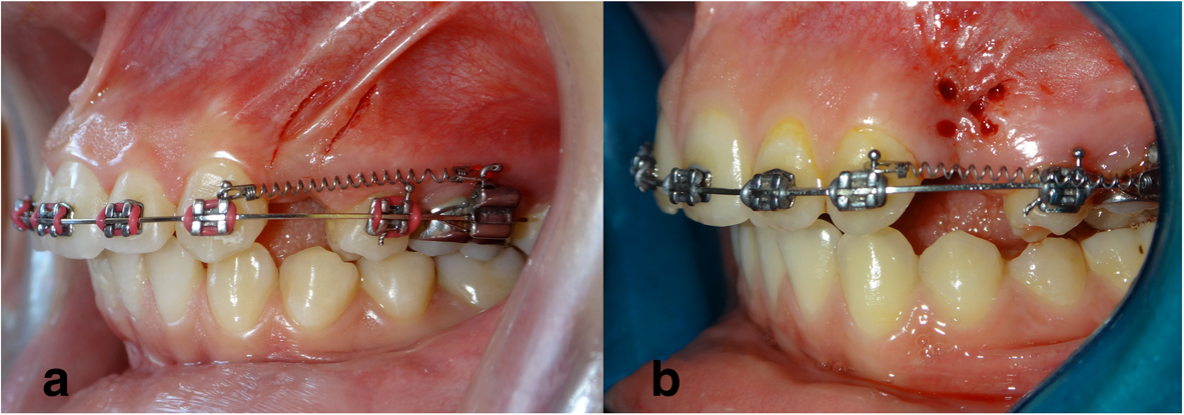
Canie retraction stage using NiTi closed coil springs immediatly following flapless corticotomy. **a**: Piezocision group. **b**: Laser-assisted flapless corticotomy group

Canine retraction was initiated immediately after the surgical intervention. 0,019 × 0,025 in. steel wires were placed for all patients and nickel-titanium closed-coil springs which extended from canine brackets to first molars bands, with 150-g force were used to retract canines (Fig. 3), the generated force was checked using force gauge (040-711-00 Dentaurum, Ispringen,Germany). Patients’ follow-up appointments were every 2 weeks to take the maximum advantage of the RAP [25]. In each appointment, force was calibrated and readjustment when necessary in order to maintain it a 150-g level during the whole retraction phase.

### Outcome measures

**Fig. 4:**
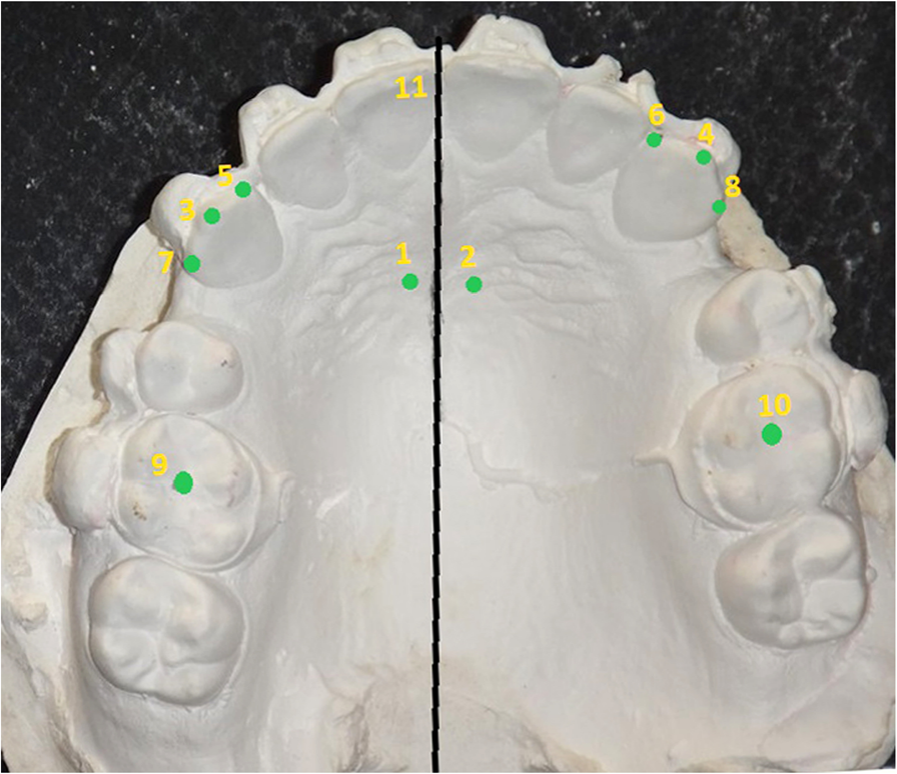
Landmarks used on plaster models for the analysis. 1: medial end of right third palatal ruga, 2: medial end of left third palatal ruga, 3: cusp tip of right canine, 4: cusp tip of left canine, 5: mesial margin of right canine, 6: mesial margin of left canine, 7: distal margin of right canine, 8 distal margin of left canine, 9: central fossa of maxillary right first permanent molar, 10: central fossa of maxillary left first permanent molar, 11: Mid-palatal suture line

**Fig. 5:**
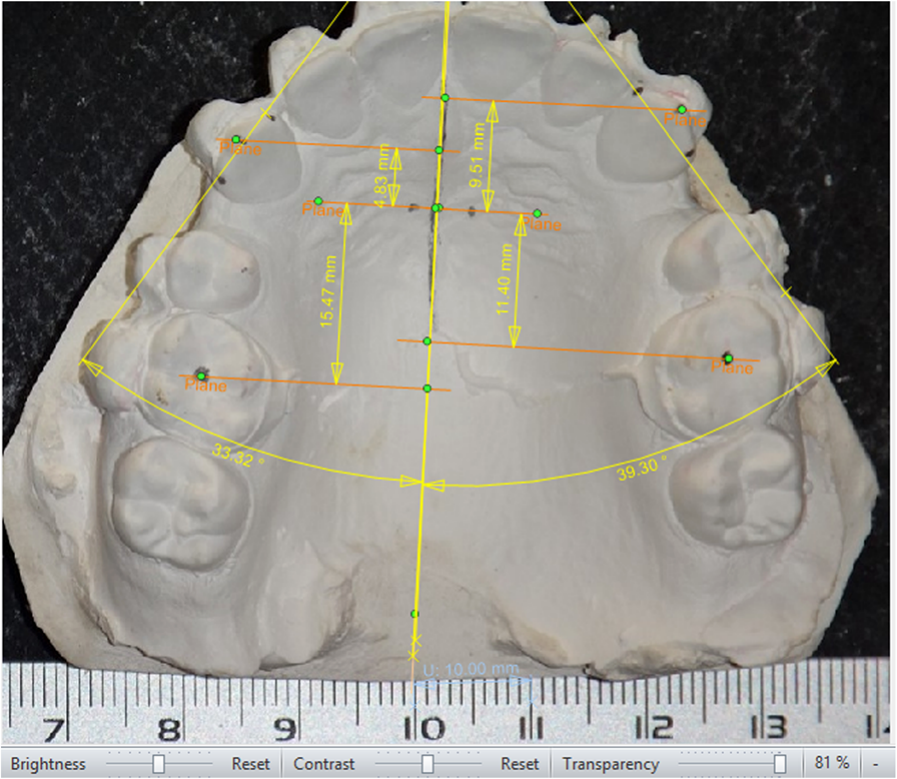
Measurements on the digital photographs with the help of AudaxCeph

The primary outcome measure was the rate of canine movement, while the secondary outcome measures were molar anchorage loss, canines’ rotation and the duration of canine retraction. Alginate impressions were taken 1 month (T1), 2 months (T2), 3 months (T3) and 4 months (T4) following the onset of canine retraction. The anterior-posterior movements of upper canines and first molars, and the changes in canines’ rotation were assessed on dental casts at four time points (T1-T4). Maxillary casts were photographed digitally with focal projection vertical to the occlusal plane and a metal millimeter ruler was placed in the same plane for the correction of magnification regarding the linear measurements. The measurements were carried out on the digital photographs using AudaxCeph® version 3.4.2.2710 (Orthodontic software suite, Ljubljana, Slovenia) with the method described by Ziegler and Ingervall [26]. This methods depends on the localization of several references points as shown in Fig. 4. Then, the following variables were measured: (1) the distance between the medial end of third palatal ruga and the cusp tip of upper canine to evaluate the anterior-posterior canine movement, (2) the distance between medial end of third palatal ruga and the central fossa of maxillary first permanent molar to evaluate the anterior-posterior molar movement, and (3) the angle between the mid-palatal suture and the line passing through the mesial and distal margins of upper canine to evalute canine rotation (Fig. 5).

The whole period of canine retraction (by months) was also recorded bilaterally in both groups, which was the period between the beginning of canine retraction until achieving Class I canine relationship. There were no outcome changes after trial commencement.

### Statistical analysis

Because of normal distribution, paired sample t-tests were used to assess the differences between the control and experimental sides within each group, while two sample t-tests were used to compare the two experimental sides from each group. To evaluate time-related changes, a one-way repeated-measures ANOVA was used. All statistical analysis were performed by one author (M.Y.H.) who was blinded to all measurements using (Minitab® Version17, StateCollege, Pennsylvania, USA).

### Error of the method

Twenty models (ten from each group) were randomly chosen. Digital photographs of casts have been repeated in the same previous manner, then all references points were reidentified and measurments were recalculated using the same software (AudaxCeph) after a four-week interval. The Intraclass correlation coefficient test (ICC) was used to determine the reproducibility of the employed method, i.e. intra-observer reliability (or random error), whereas paired t-tests were used to determine any systemtatic error. Bland & Altman plots were also used to determine the agreement between the two measurements.

## Results

**Fig. 6:**
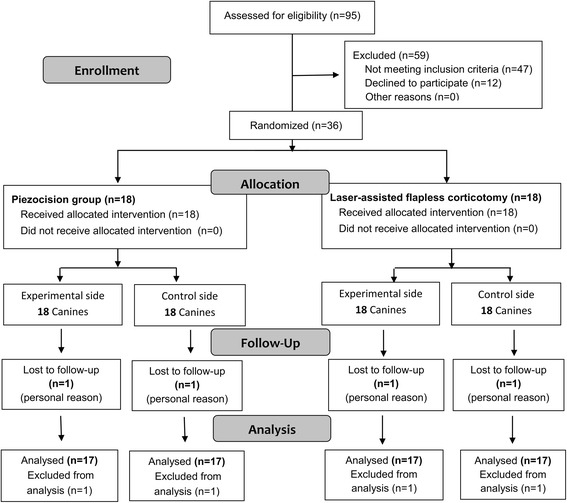
CONSORT Participants’ flow diagram

Thirty six patients were enrolled, but 2 patients (one patient in each group) were lost to follow up due to personal reasons. Therefore only 34 patients were enrolled in the statistical analysis. Patients’ allocation and follow-up is given in Fig. 6. Patient recruitment started in April 2015 and ended in February 2016, and the basic characteristics of the sample is given in Table 1.

For error of the method, Paired sample t-test results showed that there was no significant difference between the two measurements (*p* > 0.05). Therefore, systematic errors were found to be small and insignificant (Additional file 1: Table S1). The interclass correlations coefficients (ICCs) ranged from 0.989 to 0.999 which meant high reproducibility for the measurements made on plaster models (Additional file 2: Table S2). Also Bland & Altman plots demonstrated a very good agreement between the two measurements (Additional file 3: Table S3).

The rates of upper canines’ retraction were significantly higher in the experimental sides than in the control sides during the first 2 months in both groups (*p* < 0.001; Table 2). Regarding the loss of anchorage, there were no significant differences between the experimental and control sides in both groups during the four evaluation times (*p* > 0.05; Table 3). The rates of canines’ rotation were greater in the experimental sides than in the control sides in both groups during all evaluation times, however these differences were negligible and insignificant (p > 0.05; Table 4). There were no significant differences between the two experimental sides in both groups during all evaluation times for the three previous variables (*p* > 0.05; Tables 2, 3 and 4).

**Table 2:** Descriptive statistics of the canine retraction rate (mm/month) as well as the *p*-values of significance tests

Time	PG (*n* = 17)						LAFCG (*n* = 17)						PG Vs LAFCG	
	Experimental side		Control side		Mean Diff (95% CI)	*P*-Value†	Experimental side		Control side		Mean Diff (95% CI)	*P*-Value†	Mean Diff (95% CI)	*P*-Value ††
	Mean	SD	Mean	SD			Mean	SD	Mean	SD				
T0-T1 (1st month)	1.65	0.40	0.83	0.18	0.82 (0.67, 0.96)	< 0.001***	1.57	0.36	0.79	0.11	0.78 (0.62, 0.93)	< 0.001***	0.08 (− 0.19, 0.35)	0.554
T1-T2 (2nd month)	1.38	0.32	0.88	0.14	0.50 (0.36, 0.63)	< 0.001***	1.25	0.30	0.85	0.14	0.40 (0.28,0.52)	< 0.001***	0.12 (− 0.09, 0.35)	0.248
T2-T3 (3rd month)	1.10	0.29	0.98	0.22	0.11 (− 0.04, 0.26)	0.134	1.06	0.28	0.96	0.25	0.10 (− 0.06,0.27)	0.220	0.03 (− 0.18, 0.25)	0.738
T3-T4 (4th month)	0.87	0.11	0.94	0.09	−0.07 (− 0.20, 0.06)	0.231	0.89	0.16	0.90	0.16	−0.01 (− 0.16, 0.13)	0.791	− 0.01 (− 0.20, 0.17)	0.886
T0-T4	1.19	0.16	0.90	0.09	0.29 (0.12, 0.46)	0.007**	1.14	0.10	0.84	0.05	0.30 (0.14, 046)	0.006**	0.05 (− 0.14, 0.24)	0.564

†: Paired t test, ††: two-sample t test, *Significant at *P* < 0.05, **Significant at *P* < 0.01, ***Significant at *P* < 0.001, *PG* Piezocision group, *LAFCG* Laser-assisted flapless corticotomy group, *SD* standard deviation, *Mean Diff* mean difference, *CI* Confidence interval

**Table 3:** Descriptive statistics of molar movement rate (mm/month) as well as the p-values of significance tests

Time	PG (*n* = 17)						LAFCG (*n* = 17)						PG Vs LAFCG	
	Experimental side		Control side		Mean Diff (95% CI)	*P*-Value†	Experimental side		Control side		Mean Diff (95% CI)	*P*-Value†	Mean Diff (95% CI)	*P*-Value ††
	Mean	SD	Mean	SD			Mean	SD	Mean	SD				
T0-T1 (1st month)	0.65	0.26	0.77	0.24	−0.11 (−0.26, 0.02)	0.103	0.61	0.20	0.69	0.20	−0.08 (− 0.20, 0.03)	0.159	0.04 (− 0.12, 0.21)	0.589
T1-T2 (2nd month)	0.53	0.24	0.66	0.25	−0.13 (− 0.28, 0.01)	0.074	0.50	0.21	0.65	0.27	−0.14 (− 0.29, 0.01)	0.067	0.02 (− 0.13, 0.19)	0.750
T2-T3 (3rd month)	0.47	0.22	0.51	0.23	−0.04 (− 0.12, 0.02)	0.196	0.49	0.20	0.54	0.21	−0.05 (− 0.14, 0.03)	0.236	− 0.02 (− 0.18, 0.14)	0.796
T3-T4 (4th month)	0.28	0.09	0.32	0.11	−0.03 (− 0.13, 0.05)	0.322	0.32	0.22	0.33	0.19	−0.00 (− 0.15, 0.14)	0.876	− 0.03 (− 0.26, 0.19)	0.721

†: Paired t test, ††: two-sample t test, *Significant at *P* < 0.05, **Significant at P < 0.01, ***Significant at *P* < 0.001, *PG* Piezocision group, *LAFCG* Laser-assisted flapless corticotomy group, *SD* standard deviation, *Mean Diff* mean difference, *CI* Confidence interval

**Table 4:** Descriptive statistics of the canine rotation rate (degrees/month) as well as the p-values of significance tests

Time	PG (*n* = 17)						LAFCG (*n* = 17)						PG Vs LAFCG	
	Experimental side		Control side		Mean Diff (95% CI)	*P*-Value†	Experimental side		Control side		Mean Diff (95% CI)	*P*-Value†	Mean Diff (95% CI)	*P*-Value††
	Mean	SD	Mean	SD			Mean	SD	Mean	SD				
T0-T1 (1st month)	8.00	2.82	6.93	2.29	1.06 (−0.09, 2.22)	0.070	6.88	3.07	6.11	2.20	0.76 (−0.36, 1.89)	0.170	1.11 (−0.98, 3.22)	0.287
T1-T2 (2nd month)	6.54	2.88	6.19	2.43	0.34 (−0.51, 1.20)	0.403	5.82	2.26	5.59	2.53	0.23 (−0.57, 1.04)	0.544	0.71 (−1.11, 2.54)	0.433
T2-T3 (3rd month)	5.42	2.14	5.14	2.30	0.28 (−0.52, 1.09)	0.461	5.00	2.04	4.75	2.23	0.24 (−0.54, 1.04)	0.517	0.42 (−1.13,1.99)	0.580
T3-T4 (4th month)	3.22	1.44	2.82	0.61	0.40 (−0.61, 1.41)	0.355	3.39	1.62	2.53	0.99	0.86 (−1.23, 2.95)	0.316	−0.16 (−2.25, 1.91)	0.859

†: Paired t test, ††: two-sample t test, *Significant at *P* < 0.05, **Significant at *P* < 0.01, ***Significant at *P* < 0.001, *PG* Piezocision group, *LAFCG* Laser-assisted flapless corticotomy group, *SD* standard deviation, *Mean Diff* mean difference, *CI* Confidence interval

**Fig. 7:**
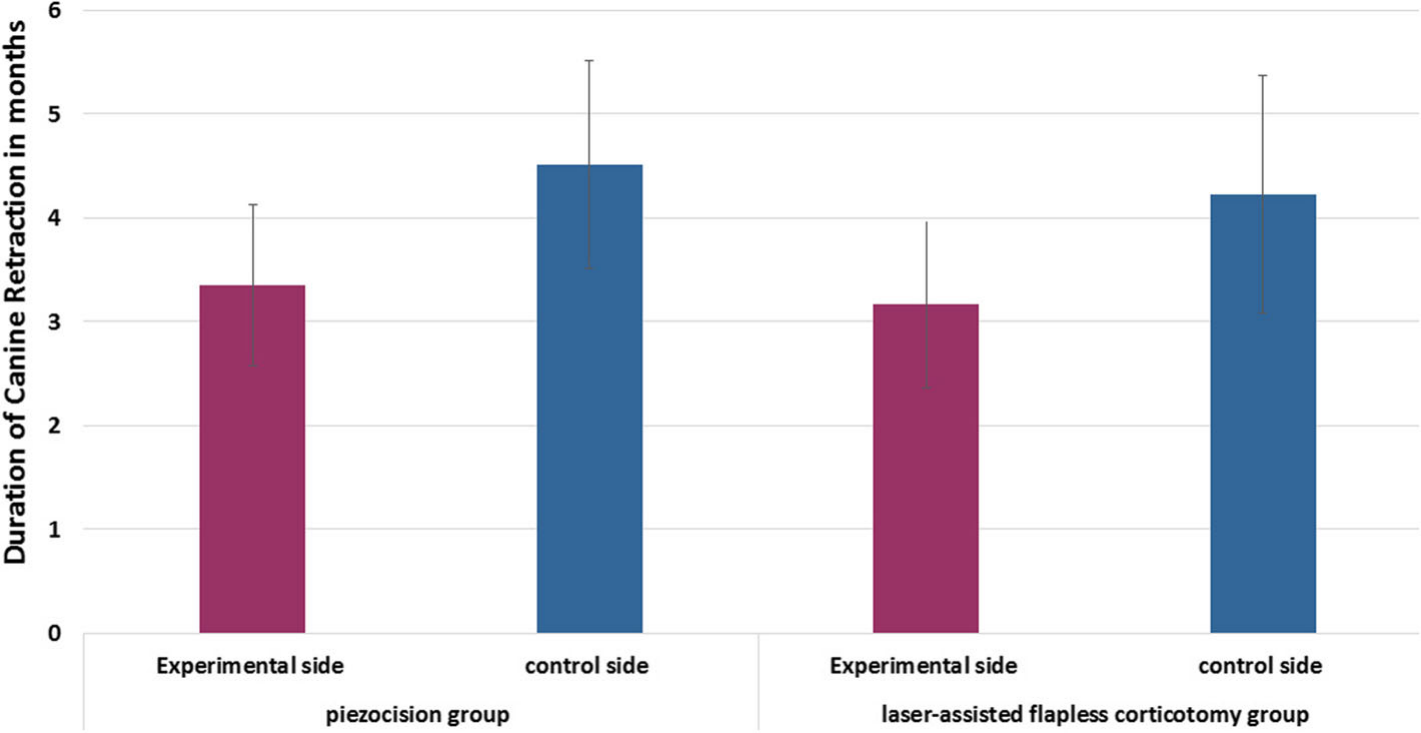
Comparison the duration of canine retraction (months) between two experimental sides in both groups

The overall duration of canine retraction was shortened significantly in the experimental side compared to the control side by a mean of 1.17 month (*p* = 0.001) in the PG and 1.05 month (*p* ≤ 0.001) in the LG whereas the difference between the two experimental sides was not significant (*p* = 0.523; Fig. 7).

The rate of canine retraction decreased significantly over time in the two experimental sides of both groups (*p* = 0.006 in the PG, *p* = 0.003 in the LG; Table 5).

**Table 5:** Descriptive statistics of the changes in the rate of canine movement over time in the experimental side for each group as well as the results of significance tests using repeated measures ANOVA and its post-hoc tests

Time	Comparisons	Piezocision group				Laser-assisted flapless corticotomy group			
		Mean Diff.	*P*-Value*	95% CI		Mean Diff.	*P*-Value*	95% CI	
				Lower bound	Upper bound			Lower bound	Upper bound
T1	T1-T2	0.21	0.013	0.06	0.35	0.46	0.062	−0.03	0.96
	T1-T3	0.41	0.045	0.01	0.81	0.74	0.023	0.16	1.31
	T1-T4	0.63	0.001	0.37	0.89	0.74	0.012	0.27	1.22
T2	T2-T3	0.20	0.141	−0.16	0.56	0.27	0.275	− 0.33	0.88
	T2-T4	0.42	0.057	0.28	0.57	0.28	0.065	−0.02	0.59
T3	T3-T4	0.22	0.107	−0.05	0.49	0.00	0.969	−0.39	0.40

*Significant at *P* < 0.05, Least Significant Difference (LSD) post-hoc tests were employed, *Mean Diff* mean difference, *CI* confidence interval

No harms were observed with piezocision and laser-assisted flapless corticotomy group during the present study.

## Discussion

According to our knowledge, this is the first trial in the literature comparing two techniques of minimally invasive corticotomy in terms of canine retraction speed, since the available evidence about the efficacy of minimally-invasive surgically-assissted orthodontics (MISAO) has been shown to be limited in a recent review [21].

Extraction of premolars was conducted in the beginning of treatment and before appliance fitting in order to allow for quicker leveling and alignment without causing additional proclination for the anterior teeth. In addition, extraction of premolars before leveling and alignment is the usual scenario for Class II division 1 patients treated at our department and this may help in evaluting the pure impact of corticotomy when performed after leveling and alighment stage instead of being performed in conjunction with extractions.

NiTi closed coil springs were used to retract canines because they generate a continuous light force and provide better oral heath compared to elastomeric chains [27]. Canine retraction was initiated immediately after the surgical intervention and patients were followed every 2 weeks instead of 4 weeks to take the maximum advantage of the RAP due to its transient nature [25]. Medial ends of the third palatal rugae were used as stable landmarks to measure the antero-posterior movements of the canine and first molar. Several studies have demonstrated that measurements taken relative to the third palatal rugae can be used as reliable as cephalometric superimposition [28].

The rates of upper canines’ retraction were significantly higher in the experimental sides than in the control sides in both groups. This acceleration might be explained by the induced RAP and to reduced alveolar bone resistance to tooth movement [15, 16]. Selective removal of alveolar bone could also stimulate the expression of inflammatory markers and increase the levels of cytokines that lead to raise the activity of osteoclasts which in turn enhance bone remodeling and accelerate tooth movement [17, 18]. Occurrence the acceleration in the first 2 months only and the gradual decrease in canine retraction speed could be attributed to the transient nature of the RAP. Wilcko et al. reported that this phenomenon had a specific pattern in its emergence and extent since it was found to start within few days following injury reaching its peak after 4 to 8 weeks and lasting for 2 to 4 months [29, 30]. However, in the current study the RAP peaked after a month and decreased dramatically at the end of the second month. The difference between the current findings and those of Wilcko et al. could be explained by the more aggressive nature of their intervention compared to that of the current trial. The rate of canine retraction in the first month was significantly higher in the piezocision side than in the control side by approximately two-fold. The rate was still 1.5 times greater in the second month. These findings agree with those of Aksakalli et al. [22] and Abbas et al. [31] who reported that piezocision was able to accelerate the rate of canine retraction significantly by 1.5-2 times during the first 3 months of tooth movement.

The rate of canine retraction was significantly faster in the laser-assisted flapless corticotomy compared to the control side by an approximately 2 times during the first month of follow-up. One recent trial conducted by Salman and Ali [13] evaluated laser-assisted flapless corticotomy and showed that there was an increase in canine retraction speed by two-fold during a six-week follow-up period. However, there were some shortcomings with their study design such as the short follow-up time as well as the poor reporting of their outcome measures.

Also the results of the current study in both groups agreed with Alikhani et al. [14] who showed that micro-osteoperforations accomplished by ‘PROPEL’ device significantly increased the speed of canine retraction during the first month of observation.

In the current study there were no significant differences between the experimental and control sides in both groups regarding the loss of anchorage which is compatible with the findings of Abbas et al. [31]. The speed of anchorage loss ranged from 0.28 to 0.65 mm/month in the piezocision group and from 0.32 to 0.61 mm/month in the Laser-assisted flapless corticotomy group; therefore, these amounts were deemed non-significant from the clinical point of view.

The rate of canines’ rotation in the surgical side in both groups was greater compared to the control side. This can be due to the greater amount of retraction in the surgical side and to low alveolar bone density caused by surgical trauma. However the increase of canine rotation was not significant in the current study, it could be explained by the conservative nature of the applied surgical interventions in the current study without producing a significant weakening of alveolar cortical bone that would allow the upper canines to rotate considerably during retraction.

There were no significant differences between piezocision and laser-assisted flapless corticotomy regarding all studied variables. This might be attributed to the minimally invasive nature of these two techniques, since they do not require flap elevation or suturing. Both techniques used innovative tools which were associated with fast healing of the alveolar bone (piezotome and ER:YAG laser). In addition, the amount of surgical injury was probably similar between the two techniques in spite of the difference in the design of incisions and bone cutting. No trial has been found in the literature comparing these two techniques, and therefore, it was difficult to compare the curring findings with any available published study.

In a recent study about patients’ and orthodontists’ perectpions towards reducing treatment time [32] it was found that orthodontists would be interested to use a modern acceleration technique if it can reduce orthodontic treatment time by 20-40%. Therefore, it seems that both piezocision and laser-assisted flapless corticotomy are possible adjunctive modalities in the acceleration of orthodontic tooth movement since they were found to shorten canine retraction time by approximately 25%.

### Limitations

Although no adverse effects were observed with the two minimally invasive corticotomy procedures in the present study, cost-benefit ratio and patient-reported outcomes have not been evaluated systematically. This study did not measure the nature of canine retraction tipping or translation. Furthermore the current trial did not evaluate sex-related possible differnces in tooth movement rate and this should be taken into account in future similar work. Therefore, the generalizability of the findings of the current trial might be representative to some extent.

## Conclusion

On the basis of the current study the following points can be concluded:

1. Piezocision and laser-assisted flapless corticotomy seemed to be effective techniques for accelerating canine retraction; canine retraction was two times faster than the conventional retraction in the first month and 1.5 times faster in the second month.
2. Piezocision and laser-assisted flapless corticotomy had no significant effects on anchorage loss or canine rotation during rapid retraction.

## Additional files

Additional file 1: **Table S1.** Assessment of the systematic error in the current study. (DOCX 19 kb)

Additional file 2: **Table S2.** Intraclass correlation coefficients of repeated measurements in the current study for the assessment of random error. (DOCX 27 kb)

Additional file 3: **Table S3.** Levels of agreement of the performed measurements in this current study according to Bland and Altman’s analysis. (DOCX 25 kb)

## Abbreviations

HILRA: Higher institution for laser research and applications
LAFC: Laser-assisted flapless corticotomy
LG: Laser-assisted flapless corticotomy group
MISAO: Minimally-invasive surgically accelerated orthodontics
PG: Piezocision group
PROPEL: A manual device could make tiny, pre-measured holes through the gingiva and alveolar bone
RAP: Regional accelerated phenomena

## References

[1] Talic NF (2011). Adverse effects of orthodontic treatment: a clinical perspective. Saudi Dent J.

[2] Segal GR, Schiffman PH, Tuncay OC (2004). Meta analysis of the treatment-related factors of external apical root resorption. Orthod Craniofac Res.

[3] Patterson BM, Dalci O, Darendeliler MA, Papadopoulou AK (2016). Corticotomies and orthodontic tooth movement: a systematic review. J Oral Maxillofac Surg.

[4] Hassan AH, Al-fraidi AA, Al-saeed SH (2010). Corticotomy-assisted orthodontic Treatment : review. Open Dent J.

[5] Alghamdi AST (2010). Corticotomy facilitated orthodontics: review of a technique. Saudi Dent J.

[6] Park YG (2016). Corticision: a flapless procedure to accelerate tooth movement. Front Oral Biol.

[7] Kim SJ, Park YG, Kang SG (2009). Effects of Corticision on paradental remodeling in orthodontic tooth movement. Angle Orthod..

[8] Dibart S, Sebaoun JD, Surmenian J (2009). Piezocision: a minimally invasive, periodontally accelerated orthodontic tooth movement procedure. Compend Contin Educ Dent.

[9] Dibart S, Surmenian J, Sebaoun JD, Montesani L (2010). Rapid treatment of class II malocclusion with piezocision: two case reports. Int J Periodontics Restorative Dent.

[10] Cassetta M, Ivani M (2017). The accuracy of computer-guided piezocision: a prospective clinical pilot study. Int J Oral Maxillofac Surg.

[11] Cassetta M, Giansanti M, Di Mambro A, Calasso S, Barbato E (2016). Minimally invasive corticotomy in orthodontics using a three-dimensional printed CAD/CAM surgical guide. Int J Oral Maxillofac Surg.

[12] Seifi M, Younessian F, Ameli N (2012). The innovated laser assisted flapless Corticotomy to enhance orthodontic tooth movement. J Lasers Med Sci.

[13] Salman LH, Ali FA (2014). Acceleration of canine movement by laser assisted flapless corticotomy [an innovative approach in clinical orthodontics]. J Baghdad College Dentistry.

[14] Alikhani M, Raptis M, Zoldan B, Sangsuwon C, Lee YB, Alyami B (2013). Effect of micro-osteoperforations on the rate of tooth movement. Am J Orthod Dentofac Orthop.

[15] Wilcko WM, Wilcko T, Bouquot JE, Ferguson DJ (2001). Rapid orthodontics with alveolar reshaping: two case reports of decrowding. Int J Periodontics Restorative Dent.

[16] Frost HM (1983). The regional acceleratory phenomenon: a review. Henry Ford Hosp Med J.

[17] Baloul SS, Gerstenfeld LC, Morgan EF, Carvalho RS, Van Dyke TE, Kantarci A (2011). Mechanism of action and morphologic changes in the alveolar bone in response to selective alveolar decortication-facilitated tooth movement. Am J Orthod Dentofac Orthop.

[18] Teixeira CC, Khoo E, Tran J, Chartres I, Liu Y, Thant LM (2010). Cytokine expression and accelerated tooth movement. J Dent Res.

[19] Vercellotti T, Podesta A (2007). Orthodontic microsurgery: a new surgically guided technique for dental movement. Int J Periodontics Restorative Dent.

[20] Dibart S (2016). Piezocision: accelerating orthodontic tooth movement while correcting hard and soft tissue deficiencies. Front Oral Biol.

[21] Alfawal AMH, Hajeer MY, Ajaj MA, Hamadah O, Brad B (2016). Effectiveness of minimally invasive surgical procedures in the acceleration of tooth movement: a systematic review and meta-analysis. Prog Orthod.

[22] Aksakalli S, Calik B, Kara B, Ezirganli S (2016). Accelerated tooth movement with piezocision and its periodontal-transversal effects in patients with class II malocclusion. Angle Orthod.

[23] Baccetti T, Franchi L, McNamara JA (2002). An improved version of the cervical vertebral maturation (CVM) method for the assessment of mandibular growth. Angle Orthod.

[24] Silness J, Loe H (1964). Periodontal disease in pregnancy. II. Correlation between oral hygiene and periodontal condition. Acta Odontol Scand.

[25] Sebaoun JD, Surmenian J, Dibart S (2011). Accelerated orthodontic treatment with piezocision: a mini-invasive alternative to conventional corticotomies. Orthod Fr.

[26] Ziegler P, Ingervall B (1989). A clinical study of maxillary canine retraction with a retraction spring and with sliding mechanics. Am J Orthod Dentofac Orthop.

[27] Dixon V, Read MJF, O'Brien KD, Worthington HV, Mandall NA (2002). A randomized clinical trial to compare three methods of orthodontic space closure. Am J Orthod Dentofac Orthop.

[28] Hoggan BR, Sadowsky C (2001). The use of palatal rugae for the assessment of anteroposterior tooth movements. Am J Orthod Dentofac Orthop.

[29] Wilcko W, Ferguson D, Bouquot J, Wilcko M (2003). Rapid orthodontic decrowding with alveolar augmentation: case report. World J Orthod.

[30] Wilcko M, Wilcko W, Bissada N (2008). An evidence-based analysis of periodontally accelerated orthodontic and osteogenic techniques: a synthesis of scientific perspectives. Semin Orthod.

[31] Abbas NH, Sabet NE, Hassan IT (2016). Evaluation of corticotomy-facilitated orthodontics and piezocision in rapid canine retraction. Am J Orthod Dentofac Orthop.

[32] Uribe F, Padala S, Allareddy V, Nanda R (2014). Patients’, parents’, and orthodontists’ perceptions of the need for and costs of additional procedures to reduce treatment time. Am J Orthod Dentofac Orthop.

