# Supplementary material for: Evaluation of piezocision and laser-assisted flapless corticotomy in the acceleration of canine retraction: a randomized controlled trial
Source: Head Face Med. 2018 Feb 17;14:4. doi: 10.1186/s13005-018-0161-9 (PMC5816528; doi:10.1186/s13005-018-0161-9)
Supplement: Supplementary file 1 — Table S1. Assessment of the systematic error in the current study. (DOCX 19 kb) [file 13005_2018_161_MOESM1_ESM.docx]

| Supplementary Table 1: Assessment of the systematic error in the current study (n=20) | | | | | |
| --- | --- | --- | --- | --- | --- |
| Variable | **Mean (SD)**  **1^ST^ measurement** | **Mean (SD)**  **2^nd^ measurement** | **Mean Difference ( 95% Cl)** | **t-value** | **P-Value** |
| Canine movement  Experimental side | 6.64 (4.04 ) | 6.63 (4.03) | 0.00 (-0.16, 0.17) | 0.092 | 0.928 |
| Canine movement  Control side | 7.53 (3.18) | 7.51 (3.37) | 0.02 (-0.20, 0.25) | 0.187 | 0.854 |
| Molar movement  Experimental side | 14.18 (2.70) | 14.18 (2.73) | 0.00 (-0.0, 0.06) | 0.150 | 0.882 |
| Molar movement  control side | 13.36 (1.94) | 13.35 (1.95) | 0.01 ) -0.04, 0.06( | 0.367 | 0.718 |
| Canine rotation  Experimental | 19.35 (12.86) | 19.10 (12.41) | 0.25) -0.12, 0.63( | 1.397 | 0.179 |
| Canine rotation  Control side | 23.30 (14.01) | 22.99 (13.96) | 0.31) -0.32, 0.95( | 1.036 | 0.313 |
| Paired t-test was used, *Significant at P<0.05, **Significant at P<0.01, ***Significant at P<0.001, SD: standard deviation, Cl: Confidence Interval. | | | | | |
