# Supplementary material for: Evaluation of piezocision and laser-assisted flapless corticotomy in the acceleration of canine retraction: a randomized controlled trial
Source: Head Face Med. 2018 Feb 17;14:4. doi: 10.1186/s13005-018-0161-9 (PMC5816528; doi:10.1186/s13005-018-0161-9)
Supplement: Supplementary file 2 — Table S2. Intraclass correlation coefficients of repeated measurements in the current study for the assessment of random error. (DOCX 27 kb) [file 13005_2018_161_MOESM2_ESM.docx]

| Supplementary table 2: Intraclass correlation coefficients of repeated measurements in the current study for the assessment of random error (n=20) | | | | |
| --- | --- | --- | --- | --- |
| Variable | **ICCs** | **95%**  **Confidence Interval** | **f-value** | **P-Value** |
| Canine movement  Experimental side | 0.996 | (0.990, 0.998) | 489.152 | <0.001 |
| Canine movement  Control side | 0.989 | (0.972, 0.996) | 177.886 | <0.001 |
| Molar movement  Experimental side | 0.999 | (0.997, 1.000) | 1646.974 | <0.001 |
| Molar movement  control side | 0.998 | (0.995, 0.999) | 1019.817 | <0.001 |
| Canine rotation  Experimental | 0.998 | (0.995, 0.999) | 965.804 | <0.001 |
| Canine rotation  Control side | 0.995 | (0.988, 0.998) | 422.513 | <0.001 |
| ICCs: Intraclass Correlation Coefficients, *Significant at P<0.05, **Significant at P<0.01, ***Significant at P<0.001. | | | | |
