# Supplementary material for: Evaluation of piezocision and laser-assisted flapless corticotomy in the acceleration of canine retraction: a randomized controlled trial
Source: Head Face Med. 2018 Feb 17;14:4. doi: 10.1186/s13005-018-0161-9 (PMC5816528; doi:10.1186/s13005-018-0161-9)
Supplement: Supplementary file 3 — Table S3. Levels of agreement of the performed measurements in this current study according to Bland and Altman’s analysis. (DOCX 25 kb) [file 13005_2018_161_MOESM3_ESM.docx]

| Supplementary table 3: Levels of agreement of the performed measurements in this current study according to Bland and Altman's analysis. | | | |
| --- | --- | --- | --- |
| Variable | **Mean (SD) of**  **difference** | **limits of agreement** | **Numbers of points out of the limits of agreement** |
| Canine movement  Experimental side | 0.01(0.37) | -0.71, 0.72)) | 2 |
| Canine movement  Control side | 0.02(0.49) | -0.94, 0.98)) | 3 |
| Molar movement  Experimental side | 0.00(0.13) | -0.26, 0.27)) | 0 |
| Molar movement  control side | 0.01(0.12) | -0.23, 0.25)) | 0 |
| Canine rotation  Experimental | 0.25(0.81) | -1.34, 1.85)) | 0 |
| Canine rotation  Control side | 0.31(1.36) | -2.35, 2.98)) | 0 |
| Bland and Altman method was used, SD: standard deviation. | | | |
